# Phase II trial of the regulatory T cell-depleting agent, denileukin diftitox, in patients with unresectable stage IV melanoma

**DOI:** 10.1186/1471-2407-11-515

**Published:** 2011-12-13

**Authors:** Sucheta Telang, Mary Ann Rasku, Amy L Clem, Karen Carter, Alden C Klarer, Wesley R Badger, Rebecca A Milam, Shesh N Rai, Jianmin Pan, Hana Gragg, Brian F Clem, Kelly M McMasters, Donald M Miller, Jason Chesney

**Affiliations:** 1 Molecular Targets Program, James Graham Brown Cancer Center, University of Louisville School of Medicine, Louisville, KY, USA; 2 Clinical Trials Office, James Graham Brown Cancer Center, University of Louisville School of Medicine, Louisville, KY, USA; 3 Department of Radiology, University of Louisville School of Medicine, Louisville, KY, USA; 4 Biostatistics Shared Facility, James Graham Brown Cancer Center, University of Louisville School of Public Health, Louisville, KY, USA; 5 Department of Surgery, University of Louisville School of Medicine, Louisville, KY, USA; 6 James Graham Brown Cancer Center, University of Louisville, 505 South Hancock Street, #424, Louisville, KY 40202, USA

## Abstract

**Background:**

We previously found that administration of an interleukin 2/diphtheria toxin conjugate (DAB/IL2; Denileukin Diftitox; ONTAK) to stage IV melanoma patients depleted CD4^+^CD25^HI^Foxp3^+ ^regulatory T cells and expanded melanoma-specific CD8^+ ^T cells. The goal of this study was to assess the clinical efficacy of DAB/IL2 in an expanded cohort of stage IV melanoma patients.

**Methods:**

In a single-center, phase II trial, DAB/IL2 (12 μg/kg; 4 daily doses; 21 day cycles) was administered to 60 unresectable stage IV melanoma patients and response rates were assessed using a combination of 2-[^18 ^F]-fluoro-2-deoxy-glucose (FDG)-positron emission tomography (PET) and computed tomography (CT) imaging.

**Results:**

After DAB/IL2 administration, 16.7% of the 60 patients had partial responses, 5% stable disease and 15% mixed responses. Importantly, 45.5% of the chemo/immuno-naïve sub-population (11/60 patients) experienced partial responses. One year survival was markedly higher in partial responders (80 ± 11.9%) relative to patients with progressive disease (23.7 ± 6.5%; *p *value < 0.001) and 40 ± 6.2% of the total DAB/IL2-treated population were alive at 1 year.

**Conclusions:**

These data support the development of multi-center, randomized trials of DAB/IL2 as a monotherapy and in combination with other immunotherapeutic agents for the treatment of stage IV melanoma.

**Trial registration:**

NCT00299689

## Background

Over 40,000 people die of metastatic melanoma each year worldwide and, in a recent review of 2,100 stage IV melanoma patients, the median overall survival was 6.2 months, with only 25.5% alive at 1 year [1]. Melanoma disproportionately affects young individuals and thus displays one of the highest "loss of potential life" rates among the adult-onset cancers (18.6 years per melanoma-related death) [2]. Current treatment options for patients with metastatic melanoma include several immunotherapeutic agents, such as high dose interleukin 2 (IL-2) [3], interferon (IFN) α-2b [4-6] and ipilimumab (an anti-cytotoxic T lymphocyte antigen-4 [CTLA4] antibody [7,8]). Unfortunately, none of these immunological strategies have improved the median overall survival of newly diagnosed stage IV melanoma patients beyond 1 year.

CD4^+^CD25^HI^Foxp3^+ ^regulatory T (Treg) cells are a subset of T cells that inhibit the activation of antigen-specific effector T cells [9,10]. Treg cells thus are an attractive cellular target for the development of novel approaches to stimulate cancer immunity [11]. Depletion of Treg cells in mice stimulates T cell-dependent immune rejection of melanoma xenografts [12-14] and Treg cells are elevated in the lymph nodes of melanoma patients [15]. Denileukin diftitox (DAB/IL2; ONTAK) is a recombinant fusion protein product of diphtheria toxin and IL-2 that selectively binds to the IL-2 receptor of cells and, following internalization, inhibits protein synthesis, causing cell death [16]. Treg cells express high levels of the alpha chain of the IL-2 receptor (CD25) and a single administration of DAB/IL2 (9 or 12 μg/kg) has been found by Curiel *et al*. to deplete Treg cells in patients with metastatic ovarian, breast or squamous cell lung carcinomas [17]. Furthermore, exposure of peripheral blood mononuclear cells to DAB/IL2 reduces the T cell suppressive activity of Treg cells *in vitro *[18]. Taken together, these studies suggest that DAB/IL2 may have clinical utility for the treatment of melanoma.

In a prior study, we examined the effect of DAB/IL2 on the peripheral blood concentration of Treg cells in 16 metastatic melanoma patients [19]. DAB/IL2 caused a transient depletion of Treg cells that coincided with the *de novo *appearance of melanoma antigen-specific CD8^+ ^T cells [19]. Although the study was not designed to assess clinical efficacy, we did observe the regression of melanoma metastases in 3 patients. In order to better define the clinical activity of DAB/IL2 against melanoma and provide rationale for randomized multi-center trials, we now have expanded this initial exploratory trial to include a total of 60 stage IV melanoma patients and will present herein the objective response rates and results of survival analyses. We find that: (i) DAB/IL2 has significant clinical activity against stage IV melanoma; (ii) lack of prior exposure to chemo/immunotherapy is associated with an increased response rate to DAB/IL2; and (iii) patients who respond live significantly longer than patients who experience progressive disease. Based on the results of this study, a new randomized multi-center clinical trial of DAB/IL2 has been initiated that will correlate Treg depletion with objective responses in chemo/immuno-naïve melanoma patients.

## Methods

### Trial design

This study was a single arm, open label phase II study of DAB/IL2 undertaken from 2007 to 2010 at the James Graham Brown Cancer Center, University of Louisville, Louisville, Kentucky. The primary objective was to determine the response rate of DAB/IL2 in stage IV metastatic melanoma patients. A secondary objective was the determination of overall survival after DAB/IL2 administration. The clinical trial registration number is NCT00299689 (at Clinicaltrials.gov).

### Patient enrollment

This clinical trial was approved by the University of Louisville Human Subjects Committee. Only patients with distant metastases from cutaneous, ocular, mucosal melanoma or melanoma of unknown primary were eligible for inclusion. All patients fulfilled the following criteria: (i) primary tumor must have been documented by histopathologic analysis; (ii) metastatic disease must have been documented by radiologic examinations (CT scan or PET scan); and (iii) disease recurrences occurring greater than 5 years after the original diagnosis must have been biopsy proven. Written informed consent was obtained from each patient prior to enrollment and the trial was conducted in accordance with the Declaration of Helsinki.

### DAB/IL2 administration

All patients were subjected to fusion FDG-PET/CT or CT imaging within 1 month prior to receiving the first dose of DAB/IL2 and within 1 month after receiving the last dose of DAB/IL2. DAB/IL2 (Eisai) was purchased through third party payers and was administered as follows: 12 μg/kg, IV over 30 min every 24 h for 4 doses (cycles repeated every 21 days; 1-4 cycles). All patients had renal function tests, blood counts, and a complete physical examination prior to each cycle of DAB/IL2. The endpoint definitions were determined from qualitative radiological assessments performed by board-certified radiologists after two cycles using the following criteria:

Clinical complete response (CR): Disappearance of all evidence of tumor. The patient must be free of all symptoms of cancer.

Partial response (PR): 30% or greater decrease in the sum of the longest diameter of target lesions, taking as reference the baseline sum longest diameter.

Progressive disease (PD): At least 20% increase in the sum of the longest diameter of target lesions, taking as reference the baseline sum longest diameter, or the appearance of new lesions and/or unequivocal progression of existing non-target lesions.

Stable disease (SD): Neither sufficient shrinkage to qualify for partial response nor sufficient increase to qualify for progressive disease, taking as reference the smallest sum longest diameter from the start of treatment.

**Figure 1:**
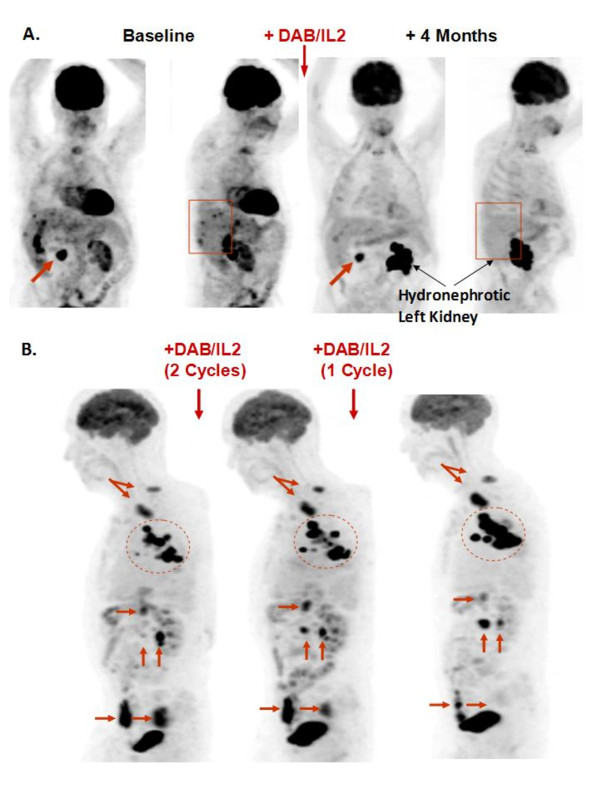
**Examples of partial and mixed responses after DAB/IL2 administration**. **A**. Anterior/posterior and lateral views of PET imaging reveal a large duodenal mass (arrow) and several hepatic metastases (box) at baseline and 1 month after the final cycle of DAB/IL2. PET imaging confirmed regression of the hepatic metastases (right panels) and a modest reduction in the duodenal mass. The increased ^18 ^F-fluorodeoxyglucose uptake in the left kidney is due to hydronephrosis which is unrelated to melanoma. **B**. After 3 cycles of DAB/IL2, this patient experienced the marked regression of a large subcutaneous and a pelvic mass (see bottom 2 horizontal red arrows just above the bladder [which normally contains tracer]) and a peritoneal mass (right vertical arrow). Simultaneously, enlarged paratracheal lymph nodes worsened (upper arrows), a large conglomeration of left axillary masses expanded (dashed circle). An inferior peritoneal mass appeared and expanded (left vertical arrow) and a superior peritoneal mass expanded and then regressed with treatment (upper horizontal arrow).

Mixed response (MR): Significant (> 30%) tumor regressions simultaneous with significant growth (> 20%) in individual tumors (see Figure 1B for an example).

Adverse events were collected by reviewing the physician dictations and nursing notes during and 1 month following the last administration of DAB/IL2.

### Statistical methods

Descriptive statistics related to patient characteristics and treatment factors were produced by outcome measurements (SD+MR+PR *vs*. PD). The Kaplan-Meier method was used to estimate the overall survival (OS). Survival differences were compared using the un-weighted log-rank test. The OS time was determined as the time from the first day of DAB/IL2 administration until death or last follow-up evaluation. We also fit the univariable and multivariable logistic regression models for the probabilities of patients with outcome SD+MR+PR about their possible predictors. All calculations were performed with SAS statistical software (SAS Institute Inc., Cary, NC).

## Results

### Baseline characteristics of study population and adverse events

We administered four daily doses of DAB/IL2 (12 μg/kg; 2-4 21 day cycles) to a total of 60 stage IV melanoma patients. The vast majority of patients enrolled in the study had metastatic melanoma involving distant organs (stage M1C; 70%) and the most commonly affected organs were the lung (47%) and liver (47%) (Table 1). 82% of patients had been treated with at least one prior systemic regimen and the majority were treated with two or more prior systemic therapies. The most common previous treatment regimens included biochemotherapy (vinblastine, dacarbazine, cisplatin, IL-2 and interferon alpha) and high dose IL-2 (Table 1). The most common adverse events reported were nausea (38%), fatigue (21%), emesis (16%), rash (15%) and chills (10%) (Table 2) and these side effects can be easily managed with symptomatic as opposed to immunosuppressive agents. Interestingly, 5% of patients reported pain associated with their tumors which may reflect inflammation caused by DAB/IL2. In this trial, only one patient developed an autoimmune disorder, vitiligo, as a result of DAB/IL2 administration. We suspect that this case of clinically insignificant vitiligo likely resulted from immune cross-reactivity against antigens expressed by both melanoma cells and melanocytes.

**Table 1:** Baseline Characteristics

	Number	Percent
**Sex (Male)**	40	67
**Ethnic Origin (Caucasian)**	59	98
**Age (Years)**	61 (median)	
**Stage**		
M1A	10	17
M1B	8	13
M1C	42	70
**Common Lesion Sites**		
Lung	28	47
Liver	28	47
Lymph Nodes	23	38
Subcutaneous	16	27
Bone	13	22
Spleen	3	5
Adrenal Glands	3	5
**Chemo- Naïve**	11	18
**Prior Chemo Exposure**	49	82
**Previous Systemic Treatments**		
0	11	18
1	18	30
2	17	28
3	9	15
4	4	7
5	1	2
**Previous Adjuvant Therapy**		
Dacarbazine/IL-2	16	27
IFN	5	8
**Previous Active Therapy**		
Biochemotherapy	26	43
High Dose IL-2	8	13
Anti-CTLA4	7	12
Temozolomide	7	12
Vaccine	7	12

**Table 2:** Adverse Events (Grade 1 + 2; ≥ 2%)

Adverse Event	Number	Percent
Nausea	23	38
Fatigue	13	21
Emesis	10	16
Rash	9	15
Chills	6	10
Back Pain	5	8
Weight Loss	4	7
Weakness	4	7
Pruritis	4	7
Poor Appetite	4	7
Shortness of Breath	4	7
Pain At Tumor Site	3	5
Insomnia	3	5
Lethargy	3	5
Rib Pain	2	3
Fever	3	5
Ascites	2	3
Cellulitis	2	3
Diarrhea	2	3
Pedal Edema	2	3
Hypotension	1	2
Cough	1	2
Whole Body Rash	1	2
Neck Pain	1	2
Presyncope	1	2
Malaise	1	2
Fluid Retention	1	2
Poor Oral Intake	1	2
Head Sweats	1	2
Diffuse Body Aches	1	2
Erythema	1	2
Abdominal Pain	1	2
Gait Imbalance	1	2
Pleuritis	1	2
Jaundice	1	2
Constipation	1	2
Nipple Irritation	1	2
Xerostomia	1	2
Chest Thrombosis	1	2
Vitiligo	1	2
Visual Disturbance	1	2
Diplopia	1	2
Perioral Numbness	1	2
Pruritus in Eyes	1	2

### Examples of clinical responses to DAB/IL2

**Figure 2:**
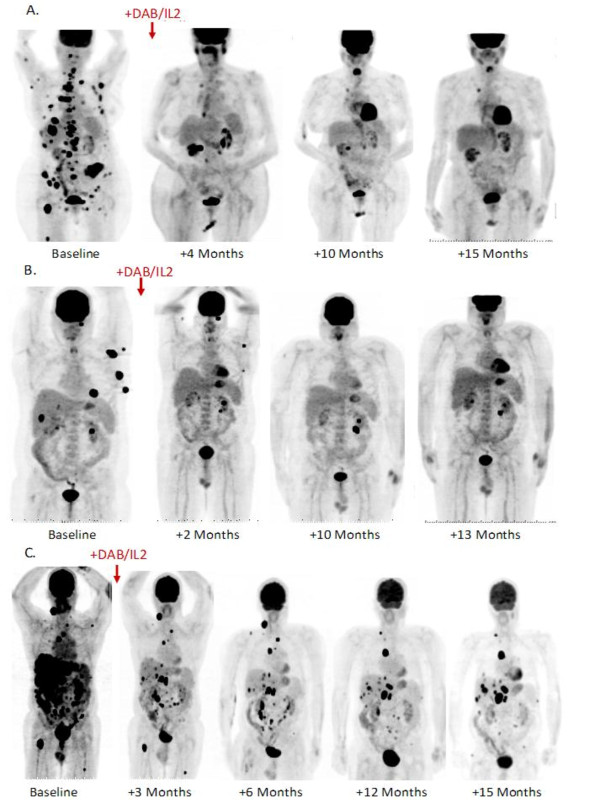
**Durable responses after DAB/IL2 administration**. On the left, baseline anterior/posterior views of FDG-PET imaging reveal multiple FDG-avid melanoma metastases in three clinical examples: **A**, 78 year-old female; **B**, 47 year-old male; and **C**, 62 year-old male. After DAB/IL2 administration, we observed a clinically significant and durable reduction in tumor burden, including hepatic metastases, pulmonary nodules, metatastic lymph nodes and subcutaneous nodules in all three patient examples.

We observed several examples of partial and mixed responses which are typical of immunotherapeutic agents. For example, an 82 year-old male developed multiple hepatic metastases (red box, second panel; Figure 1A) and a large duodenal mass (red arrow, first panel, Figure 1A) which caused significant nausea, vomiting and weight loss. After four cycles of DAB/IL2, he experienced the complete regression of his hepatic metastases confirmed by FDG-PET imaging and resolution of his symptoms but only a modest reduction in his duodenal mass (compare Baseline to +4 Months, Figure 1A; the increased ^18 ^F-FDG uptake in the left kidney is due to hydronephrosis which is unrelated to melanoma). Next, an 83 year-old male received three cycles of DAB/IL2 and experienced marked regression of a large subcutaneous mass, a pelvic mass (Figure 1B, see bottom 2 horizontal red arrows just above the bladder [which normally contains tracer]) and a peritoneal mass (Figure 1B, right vertical arrow). Simultaneously, a large conglomeration of left axillary masses expanded (Figure 1B, dashed circle), paratracheal lymph nodes worsened (Figure 1B, upper arrows) and a peritoneal mass appeared and expanded with treatment (Figure 1B, left vertical arrow). This is a typical clinical example of a mixed response to DAB/IL2. A 78 year-old female experienced a dramatic reduction in metastases involving the liver, lung and bone that has persisted for 15 months with the exception of a single small right paratracheal lymph node (Figure 2A). A 47 year-old male who had previously progressed through high dose IL-2, biochemotherapy and several experimental agents also had a marked global reduction in hepatic, lung and subcutaneous metastatic burden (Figure 2B). As a final clinical example, a 62 year-old male who progressed after receiving anti-CTLA4 and experienced debilitating right upper quadrant pain, nausea/vomiting and fatigue associated with widespread hepatic metastases experienced a substantial partial response that was durable for at least 15 months (Figure 2C). These examples of partial but durable clinical responses to DAB/IL2 are suggestive of an immunotherapeutic mechanism of action for DAB/IL2.

### Objective clinical response rates based on qualitative radiological assessments

**Figure 3:**
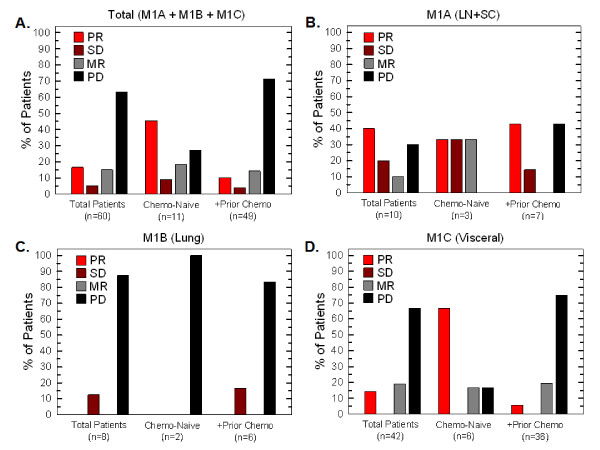
**A. Increased partial responses to DAB/IL2 noted in chemo/immuno-naïve and stage M1A melanoma patients**. Sixty stage IV melanoma patients were administered DAB/IL2 and the objective response rates were determined using qualitative radiological assessments of FDG-PET and CT imaging. Whereas 16.7% of all treated patients experienced partial responses, 45.5% of the chemo/immuno-naïve patients experienced partial responses (**A**). Response rates were calculated based on sub-stage of melanoma. Whereas stage M1A (lymph node, skin and subcutaneous) (**B**) melanoma patients experienced the highest total objective response rate, stage M1B patients had no partial responses (**C**) and chemo/immuno-naïve patients with visceral metastases (stage M1C) had a > 60% partial response rate (**D**).

We compared baseline FDG-PET and/or CT imaging to follow-up scans using qualitative radiology assessments which approximate Response Evaluation Criteria in Solid Tumors (RECIST) criteria [20]. Importantly, any new lesions that were identified would automatically signify progression. Figure 3A illustrates the following response rates: partial response (PR), 16.7% (10/60); stable disease (SD), 5% (3/60); mixed response (MR), 15% (9/60); and progressive disease (PD), 63.3% (38/60). We observed a marked improvement in the response rates of the 11 chemo/immuno-naïve patients: partial response (PR), 45.5% (5/11); stable disease (SD), 9.1% (1/11); mixed response (MR), 18.2% (2/11); and progressive disease (PD), 27.3% (3/11) (Figure 3A). Univariable and multivariable logistic modeling revealed a statistically significant increase in patients experiencing clinical benefit (PR+SD+MR) in the chemo/immuno-naïve population (*p *values = 0.011 and 0.017, respectively). We did not observe a decrease in response rate from prior exposure to IL-2 (*p *value = 0.389, univariable logistic model) which had been anticipated given the potential for cross-reactivity of antibodies between recombinant IL-2 and DAB/IL2 (*data not shown*). Stage IV melanoma is sub-classified into M1A (metastases to skin, subcutaneous tissues or distant lymph nodes; normal LDH), M1B (lung; normal LDH) and M1C (other visceral sites or distant metastases at any site; normal LDH; or any distant metastases; elevated LDH). We found that the partial response rate was highest in M1A patients (40%; Figure 3B and Table 3) and univariable logistic modeling indicated that the combined PR+SD+MR rate in the M1A population was higher than in the M1B population (*p *value = 0.028) and the M1C population (*p *value = 0.044). However, within the chemo/immuno-naïve population, the M1C patients experienced the greatest partial response rate (66.7%; Figure 3D). These data suggest that patients with the worst prognosis (*i.e*. M1C) seem to respond to DAB/IL2 at least as well as those with higher survival odds. No M1B patients had a partial or mixed response and only one did not progress (12.5%; Figure 3C). Last, although only two mucosal and two ocular melanoma patients were enrolled, we did observe 2/2 mixed responses and 1/2 mixed response in this small population, respectively (*data not shown*).

**Table 3:** Stages and Prior Systemic Therapies of DAB/IL2 Responders

Stage	Age	Prior Systemic Therapy	# Cycles	Response
M1A (7/10)	59	None	4	PR
	78	None	2	SD
	83	None	2	MR
	64	ADI, BCT, *α*CTLA4, VAC	4	PR
	60	AIFN, BCT, TEM	4	PR
	45	ADI, IFN, VAC	4	PR
	75	AIFN, BCT	4	SD
M1B (1/8)	67	ADI	4	SD
M1C (14/42)	78	None	2	PR
	83	None	3	PR
	82	None	3	PR
	61	None	4	PR
	59	None	4	MR
	47	BCT, HDIL2, TKI258	3	PR
	62	*α*CTLA4	4	PR
	55	BCT, HDIL2, TKI258	4	MR
	70	ADI, BCT	2	MR
	30	BCT	4	MR
	30	BCT, HDIL2, *α*CTLA4, TKI258	2	MR
	45	BCT	4	MR
	61	BCT	3	MR
	58	ADI, VAC	4	MR

Abbreviations: *ADI *adjuvant dacarbazine/low-dose IL-2; *BCT *biochemotherapy; *αCTLA4 *anti-cytotoxic T lymphocyte Antigen 4 antibody; *VAC *peptide vaccines; *TEM *temozolomide; *IFN *interferon; *AIFN *adjuvant interferon; *HDIL2 *high-dose IL-2; *TKI258 *tyrosine kinase inhibitor 258

### Survival analyses

**Figure 4:**
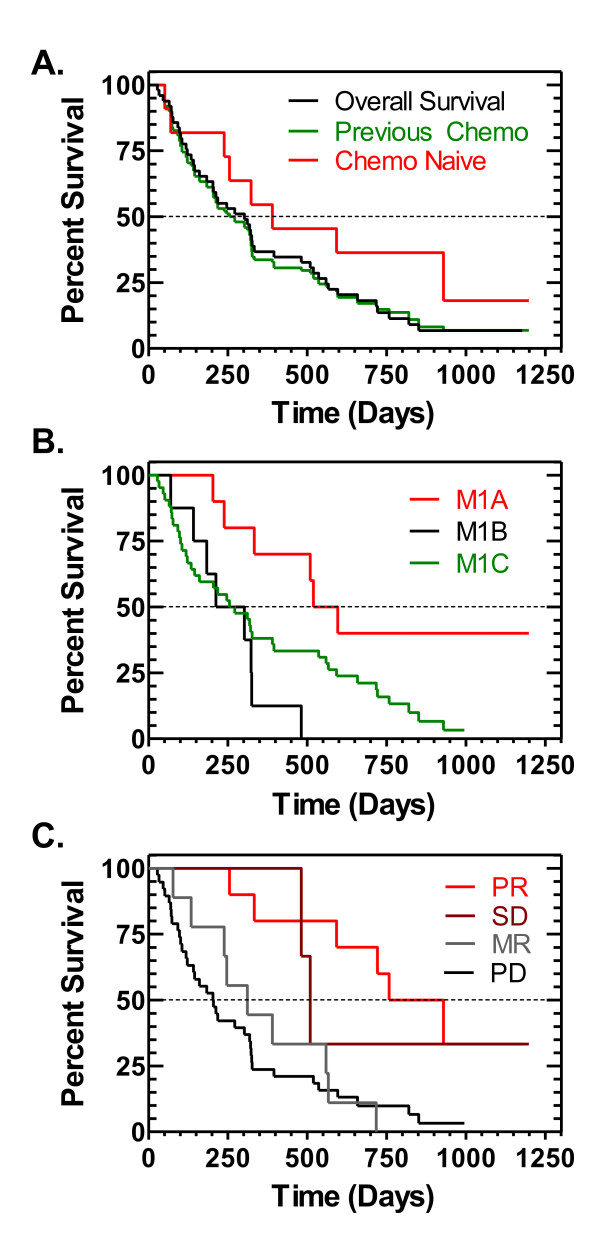
**High overall survival in chemo/immuno-naïve, stage M1A and partial responders to DAB/IL2**. Median overall survival of these patients was determined using the Kaplan-Meier method and stratified based on prior treatment exposure (**A**), stage (M1A-C) (**B**) and response rate (**C**).

The median follow-up day from the first day of DAB/IL2 was 315 days (range from 28 to 1,198 days) for all patients and 995 days for seven patients who were alive at the time of the last follow-up (range from 631 to 1,198 days). The 1, 2, 3 and 4 year overall survival percentages were 40.0%, 17.9%, 9.2% and 9.2%, respectively. Although there appeared to be a trend towards improved overall survival in the chemo/immuno-naïve population (Figure 4A), the un-weighted log-rank test did not reveal a statistically significant difference (*p *value = 0.140). However, the overall survival probability was significantly higher in the patients in stage M1A compared to those in stage M1B (*p *value = 0.001), stage M1C (*p *value = 0.019) and combined stage M1B+M1C (*p *value = 0.010; Figure 4B) and the patients with a PR had a statistically significant longer overall survival time than those with the outcome PD (*p *value < 0.001; Figure 4C). Last, there appeared to be a trend towards decreased overall survival at year 2 in patients who had been previously administered recombinant IL-2 (*data not shown*), however this was not statistically significant (*p *value = 0.178).

## Discussion

This single-center, exploratory trial demonstrated that DAB/IL2 has significant clinical activity in stage IV melanoma patients. The finding that partial responses to DAB/IL2 were associated with longer overall survival provides preliminary rationale for clinical trials in which patients are randomized to DAB/IL2 or FDA-approved agents for stage IV melanoma (*i.e*. IL-2, dacarbazine, ipilimumab or vemurafenib). Importantly, the 1 year median overall survival of 40% in this predominantly pre-treated stage IV melanoma population compared favorably to the historical 1 year overall survival of 25.5% [1]. Additionally, the observed immunotherapy-like mixed responses (*e.g*. Figure 1B) and partial but durable responses (*e.g*. Figure 2) coupled to the known lack of cytotoxicity of DAB/IL2 to human melanoma cells implies that the clinical activity of DAB/IL2 may rely in part on the known Treg-depleting effects of DAB/IL2 [19]. However, we should note that one prior study did not detect a depletion of Treg cells after DAB/IL2 administration which may due to differences in their Treg cell measurement methodologies or the effects of prior treatments on the Treg-depleting activity of DAB/IL2 (discussed in [21].)

Based on the high response rates in the chemo/immuno-naïve patients (Figure 3A), a new multi-center, sponsored phase II trial of DAB/IL2 in chemo/immuno-naïve patients that relies on CT imaging and immune related response criteria was initiated in Summer 2010. This trial has been powered to correlate the clinical effects of DAB/IL2 with the depletion of peripheral blood Treg cells. CD8^+ ^T cell infiltration into tumors and, perhaps most importantly, HLA class I expression of the melanoma cells, will be assessed by immunohistochemistry of tumors from patients who agree to undergo biopsies. We postulate that the patients who have the greatest Treg cell depletion may experience more clinical responses but that certain melanoma metastases will nevertheless grow due to immune escape through decreased HLA class I antigen expression and/or decreased melanoma antigen expression.

The failure to mount effective immunity against melanoma cells likely results from a combination of attenuated priming of naïve CD4^+ ^T cells due to suppression of antigen presentation by dendritic cells coupled to selection for loss of class I major histocompatibility complex (MHC) expression in proliferating melanoma cells, negative regulation by surface CTLA4 in CD4^+ ^and CD8^+ ^effector T cells and the direct suppression of these cells by Treg cells, among other factors [22]. We now have the clinical tools to simultaneously activate dendritic cells both *ex vivo *and *in situ *(*e.g*. with granulocyte macrophage -colony stimulating factor, GM-CSF), to upregulate the expression of class I MHC in a subset of melanoma cells with recombinant interferons, to block the interaction between CTLA4 and its ligands, CD80 and CD86, with humanized antibodies (*e.g*. ipilimumab), to transiently deplete regulatory cells and stimulate the peripheral blood concentration of antigen presenting cells with DAB/IL2, and to introduce peptide antigens that contain well-defined T cell epitopes (*e.g*. gp100). While such combinations of immunotherapeutic agents certainly have the potential to cause chronic or potentially life-threatening autoimmunities, we believe that the < 1 year median overall survival of stage IV melanoma patients supports an acceptable risk:benefit ratio for testing in clinical trials.

## Conclusions

We conclude that DAB/IL2 has significant clinical activity in unresectable stage IV melanoma patients. We anticipate that the new phase II clinical trial of DAB/IL2 will yield definitive objective response rates that will correlate with Treg cell depletion and that the efficacy of this agent will be improved through the testing of rational immunotherapeutic combinations.

## Competing interests

The authors declare that they have no competing interests.

## Authors' contributions

All authors have read and approved the final manuscript. The specific contributions of each author are: ST did the majority of the clinical data collection and analysis; MAR collected adverse events and response data; ALC assisted in the survival data collection; KC provided clinical nursing support; ACK collected the death rate data by month; WRB coordinated the statistical analyses; RAM analyzed the presented PET figures; SNR and JN conducted the statistical analyses; HG provided the regulatory support for the trial; BFC assisted in response data analysis; KMM and DMM provided surgical and medical oncology care for the patients and were sub-investigators on the clinical trial; JC conceived, designed and directed the entire study, interpreted all data and wrote the manuscript.

## Pre-publication history

The pre-publication history for this paper can be accessed here:

http://www.biomedcentral.com/1471-2407/11/515/prepub
